# The Study of Medicine

**Published:** 1911-09-02

**Authors:** 


The Hospital

A Journal of

The Medical Sciences and Hospital Administration.

New Series. No. 240, Vol. IX. [No. 1312, Vol. L.] SATURDAY, SEPTEMBER 2, 1911.

### EDUCATIONAL NUMBER.

### THE STUDY OF MEDICINE.

By a HOSPITAL PHYSICIAN.

The commencement of a new study may be compared in some measure to the entering of a country hitherto unvisited. Fresh scenes are continually presenting themselves to the view. These scenes give rise to different emotions, according to the temperament of the observer: among travellers we have the tramp, the combatant—*i.e.* the one ready to decry everything which does not fit in with his own ideas—and the collector, he who is armed with a notebook to record facts. Similarly among those who are required by the nature of their future calling to employ their minds in the acquisition of knowledge mainly from books, are the tramp, the fighter—or we may call him the reasoner—and the collector. The tramp finds new ideas of interest, but when they have lost their novelty he may have no further use for them. The reasoner may find his chief interest in new ideas lies in a desire to destroy them. The collector looks upon all facts and ideas as items of knowledge to add to his mental museum.

### The Tramp and the Collector.

Needless to say, few minds present unmixed the qualities of the tramp, the reasoner, or the collector, but the great majority of minds have characteristics which would place them mainly under one of the headings. The particular branch of the calling which may be followed in later life will probably depend upon the qualities of the mind. He who has the qualities mainly of the tramp and the reasoner will probably make a better investigator than he who possesses the qualities of the reasoner and the collector. The tramp is the man of artistic temperament who more than others is likely to see visions. This, if combined with a keen critical faculty, is the most valuable type of mind for investigation.

### The Practical Aspect.

On the other hand, the collector of facts who has also the power to reason out the value of facts and ideas is the man to deal with the affairs of everyday life. He is the practical man. Educational methods are a frequent basis for discussion. Ideas upon the most suitable method for training the mind are many and various. In the past there can be little doubt that those responsible for educating the young looked upon education mainly as the acquisition of facts and opinions. And the examination papers issued under the auspices of various examining bodies favoured this view. The minds of learned men themselves—the teachers of others—seemed to be swamped by facts. We once heard a learned judge, himself a scientist as well as a judge, say that the "majority of scientific writing was like a mass of facts hurled at the reader." Where the view that education consists mainly in the acquisition of facts or of opinions, no room is left for critical examination of the relative value of facts or of opinions based upon them.

### Untested Knowledge.

The well-known saying is that "knowledge is power," but the knowledge must be sound and one must be competent to use it. There is no more helpless member of society than he whose mind is stored with facts and ideas the value and truth of which he is never able to put to the test. A learned judge has been quoted; we may also quote a learned professor, but a practical professor. He once remarked that savage man is in some ways stronger than the educated civilised man. The savage man, so far as observations are concerned which are required for the daily occupations of life, has proved for himself the value of such observations. The educated civilised man may meet with circumstances in which he may find the knowledge at his command which he had deemed suited to the case to be entirely inadequate. His second-hand knowledge has been based upon faulty observations.

### The Value of Original Observation.

In the study of medicine, possibly more than in some forms of study, the curriculum is so arranged that the student can prove the truth of observations for himself. The knowledge of man himself may be said in part to be finite, in part infinite. So far as the material details of his body are concerned—at least in their naked eye appearances—the knowledge may be said to be finite. Of the finite knowledge exceptional industry may enable a student to become almost a complete master by handling and seeing every detail for himself. When, however, not the material parts of the the body, but their functions are considered, here it must be said that knowledge is virtually infinite. The depths of the working of some parts of our nature are apparently as unfathomable as life itself. Yet, needless to say, in spite of the marvellous intricacies of our being, much is known of the various processes at work within us.

### Anatomy and Physiology.

Little of this knowledge, however, the student will be able to prove for himself. But though his knowledge concerning these processes will be mainly second-hand knowledge, his study of them will afford useful training for the mind. The study of anatomy is a study suited to the collector of facts. The study of physiology is most adapted to the reasoner. In acquiring a knowledge of physiology the student will see how step by step certain conclusions have been arrived at. He will see how different workers have arrived at different conclusions, and how the truth of either of those conclusions will depend upon the respective value of observations or accuracy of reasoning. It may be obvious that both are wrong, and some new path of investigation on which to travel may suggest itself to that type of mind which above has been called the mind of the tramp. Here it may be mentioned that the mind of the tramp, pure and simple, is not a type of mind to be desired. It is a type of mind which dislikes effort. Allied with reasoning power, however, its character is not so much a desire " to seek pastures new " as to look out for " fresh worlds to conquer."

### The New Domain of Clinical Work.

When the first part of the medical career is over, and the body, not in health but in disease, is the subject for study, how many fields still remain to be conquered will become very apparent. The surgeon occupies the position of pre-eminence in the practice of medicine of the present day if the word medicine is used in its wide sense. The deeds of the surgeon appeal strongly to the imagination of the public. Possibly the surgeon we shall always require to have with us. Yet to allow something to attack us and to remove it forcibly from our bodies is not a sequel of events that appeals to the highest ideal of scientific medicine. The aim of medicine is to learn the nature of disease and how to prevent it. Medicine occupies this peculiar position—the more successful it becomes in fulfilling its aims the less work there is for its followers. As the health of the community improves, the less the need for entrants into the medical profession.

### Faulty Methods of Teaching.

In closing it may be suggested that there is possibly scope for improvement in the system of teaching medicine. Anatomy, as at present taught, is for the majority of students very uninteresting. To use a slang expression, it may be described as a " dull grind." The method of study may be useful as a training for the memory and in inculcating attention to accuracy of detail, but as regards the value of the knowledge itself it is to be feared this is comparatively small. In spite of the study of anatomy possibly being of some service in training the memory, it is probable that by the time the student commences to learn operative surgery he has forgotten the greater part of his anatomy. The study of anatomy could be made much more interesting and the practical value of some knowledge of anatomy would be made evident if a certain amount of study of surgery or of medicine could be combined with it. For example, the nature of deformation following paralysis of muscles resulting from nerve lesions would render the necessity for the knowledge of the nerve-supply of muscles intelligible, and the symptoms produced by aneurism of the aorta in various parts of the arch make the varied relations of the aorta interesting. In the same way the study of the physiology of various glands or of the nervous system might be lightened by reference to various diseases. It is not suggested that such a method as this should be extensively introduced. Yet the association of the study of the normal with the abnormal might be employed sufficiently to increase interest and indicate the practical bearing of knowledge in the first and second years of study. At present the student has to exercise the belief that the knowledge he is endeavouring to acquire may be useful. He is unable to form an estimate of its possible usefulness.

Upon entering the path which is to carry him through the domain of medical study, the student will at first be worried by the immensity of the new country that lies before him. If, however, he bears in mind the fact that his work is not to be pioneering or exploring so much as surveying what has already been done, he will be encouraged to proceed with energy tempered by caution and discretion, until he arrives at the stage when he himself can take his part, thoroughly equipped by knowledge and skill, in exploiting the unworked resources that are still to be found in that vast domain.

